# Low molecular weight heparin monotherapy for recurrent abortion with antiphospholipid system: A protocol of a systematic review

**DOI:** 10.1097/MD.0000000000014619

**Published:** 2019-02-22

**Authors:** Lei Ni, Xue-qian Sun, Dong-xu Zhao, Zi-wei Zhu

**Affiliations:** a Department of Hematology; b Department of Immunology; c Department of Gynecology, First Affiliated Hospital of Jiamusi University, Jiamusi, China.

**Keywords:** antiphospholipid system, efficacy, low molecular weight heparin, recurrent abortion, safety, systematic review

## Abstract

**Background:**

Previous clinical studies reported low molecular weight heparin (LMWH) monotherpay has been utilized for the treatment of recurrent abortion (RCA) with antiphospholipid system (APS). However, its efficacy is still inconclusive. This systematic review aims to assess its efficacy and safety for patients with RCA and APS.

**Methods:**

A systematic literature search for article up to February 2019 will be conducted in 9 databases: Cochrane Library, EMBASE, MEDILINE, Cumulative Index to Nursing and Allied Health Literature, Allied and Complementary Medicine Database, Chinese Biomedical Literature Database, China National Knowledge Infrastructure, VIP Information, and Wanfang Data. Inclusion criteria are randomized control trials of LMWH monotherpay for patients with RCA and APS. The Cochrane risk of bias tool will be used to evaluate the methodological quality for each qualified study. The summary results will be showed by using fixed-effects and random-effects models for pooling the data based on the heterogeneity of included studies.

**Results:**

This systematic review will assess the clinical efficacy and safety of LMWH monotherpay in treating RCA with APS. The primary outcome is pregnancy loss. The secondary outcomes include frequency of preterm delivery, live birth rates, maternal and fetal complications, as well as adverse events.

**Conclusion:**

The findings of this study will summarize the present evidence to judge whether LMWH monotherpay is an effective therapy for patients with RCA and APS.

**Dissemination and ethics:**

The findings of this study will be published by through peer-reviewed journals. This study does not needs ethic documents, because it will not analyze individual patient data.

**Systematic review registration:**

PROSPERO CRD42019121064.

## 1 Introduction

Antiphospholipid syndrome (APS) is an autoimmune, multisystemic disorder.^[1,2]^ It is diagnosed according to the present clinical and laboratory classification criteria,^[3,4]^ and is widely recognized as a risk factor for a variety of pregnancy complications.^[5,6]^ These complications often consist of fetal loss, miscarriage, intrauterine growth restriction, preeclampsia, fetal death, and preterm delivery.^[7–9]^ Previous study has reported that APS can account for 15% recurrent abortion (RCA).^[10–11]^ The incidence of APS is about 5 individuals per 100,000 patients/y, and its prevalence is about 40 to 50 patient every 100,000 subjects.^[12]^

A numerous clinical trials have reported that low molecular weight heparin (LMWH) has been used for the treatment of recurrent abortion (RCA) with antiphospholipid system (APS), and have achieved promising outcomes.^[13–23]^ Although a previous systematic review has addressed for assessing the efficacy of the combination of heparin and aspirin compared with aspirin monotherapy in pregnant women with RCA and APS in 2010,^[24]^ several trials have been published after that study.^[13,14]^ Moreover, no systematic review specifically investigated the efficacy and safety of LMWH monotherapy (LMWH) compared with any other therapies for the treatment of RCA with APS. Therefore, in this study, we will assess the efficacy and safety of LMWH monotherapy for treating RCA with APS, which is totally different from the previous published study.^[24]^

## 2 Methods

### 2.1 Ethics statement

This study does not needs ethic approval, because it will not analyze individual patient data.

### 2.2 Objective

This systematic review and meta-analysis aims to evaluate the efficacy and safety of LMWH monotherapy for treating RCA with APS.

### 2.3 Study registration

This protocol has been designed and reported in accordance with the Preferred Reporting Items for Systematic Reviews and Meta-Analysis Protocol (PRISMA-P) statement guidelines,^[25]^ and it has been registered in PROSPERO (CRD42019121064).

### 2.4 Inclusion and exclusion criteria

All included studies must meet the following criteria:

(1) randomized controlled trials (RCTs);
(2) Study examined patients with diagnosed of RCA and APS, who received LMWH monotherapy compared with any other therapies, except the LMWH.
(3) Patients with RCA and APS, regardless race, sex, and age will be included.

The exclusion criteria are as follows: duplicated publications; non-clinical trials, case reports, case series, observational studies, qualitative studies, letters, comments, non-RCTs, and quasi-RCTs; LMWH was used in both experimental and control groups; combination of LMWH with other treatments.

### 2.5 Intervention type

Intervention of any forms of LMWH monotherapy will be included. There will be no limitations of forms, dosage, frequency, duration of LMWH monotherapy. Control therapy can be any treatments, but not any forms of LMWH monotherapy.

### 2.6 Outcome measurements

The primary outcome includes pregnancy loss. The secondary outcomes consist of frequency of preterm delivery, live birth rates, and maternal and fetal complications. In addition, adverse events will also be assessed.

### 2.7 Search strategy

Nine databases of Cochrane Library, EMBASE, MEDILINE, Cumulative Index to Nursing and Allied Health Literature, Allied and Complementary Medicine Database, Chinese Biomedical Literature Database, China National Knowledge Infrastructure, VIP Information, and Wanfang Data will be retrieved up to the February 2019. In addition, we will also search the reference lists of relevant reviews and included studies. The detailed search strategy of Cochrane Libaray is described in Table 1. Similar detailed search strategies will be applied to other databases.

**Table 1:**
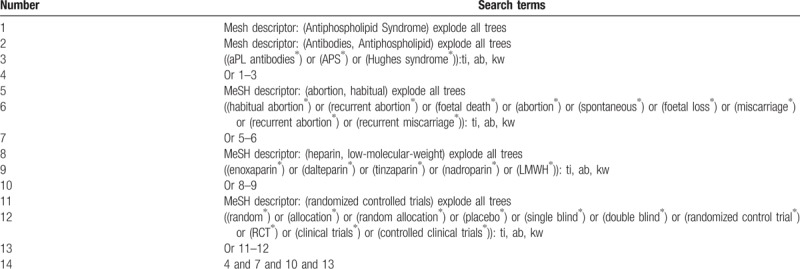
Search strategy applied in Cochrane Library database.

### 2.8 Study selection

**Figure 1:**
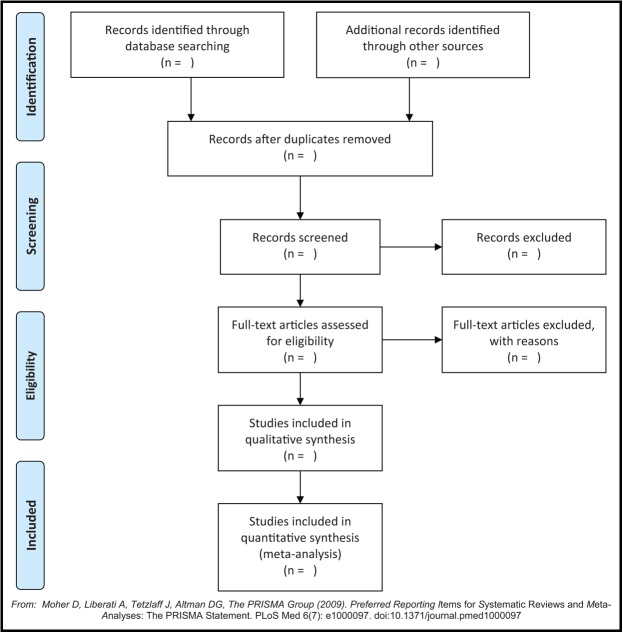
Flowchart of study selection process.

Two authors will independently scan the titles and summary of potential studies according to the predefined eligibility criteria. If studies meet the initial inclusion criteria, full-texts will be further reviewed to make sure that they meet all the eligibility criteria. All procedures of study selection follow the PRISMA flowchart, which is presented in Fig. 1. If it meets any divergences, they will be solved by consulting another experienced author.

### 2.9 Data extraction and management

Two authors will independently carry out the data extraction using predefined standard data collection form. The following data will be extracted: general study information (first author, published year, and country); patient and disease characteristics (sample size, age, sex, and race, diagnostic criteria, inclusion and exclusion criteria); study methods (randomization, allocation, blinding, and any other risk bias); interventions (types, dosages, frequencies, durations of interventions), and outcomes (primary and secondary outcomes, adverse events). Any discrepancies will be resolved by consulting another experienced author. All extracted data will be inputted into RevMan 5.3 (Cochrane Community, London, UK) software for analysis.

### 2.10 Dealing with missing data

If the data are missing, or insufficient or unclear, we will contact original authors to request those data. If those data are not obtainable, we will just analyze the available data, and will also discuss the potential impact of missing data.

### 2.11 Risk of bias assessment

We will use Cochrane Handbook for Systematic Reviews of Interventions Tool as standard criteria for judging the risk of bias for each qualified trial.^[26]^ All the process of risk of bias assessment will be performed by 2 independent authors. Any disagreements will be settled by consulting another author.

### 2.12 Statistical analysis

All outcome data will be analyzed by using RevMan 5.3 software. All the continuous data will be expressed as mean difference or standardized mean difference and 95% confidence intervals (CIs), while all the dichotomous data will be expressed as risk ratio and 95% CIs.

*I*^*2*^ test will be utilized to assess the heterogeneity. A fixed-effect model will be used to pool the data, and meta-analysis will be conducted, if acceptable heterogeneity is detected (*I*^*2*^ < 50). Otherwise, a random-effect model will be used if substantial heterogeneity is found (*I*^2^ ≥ 50). Under such situation, subgroup analysis will be carried out according to the different treatment types, control therapies, and outcome measurements. A narrative summary will be reported if heterogeneity is still significant after the subgroup analysis, and data will not be pooled, as well as the meta-analysis will not be carried out.

Sensitivity analysis will be performed to detect the robustness of pooled data by removing low quality of studies. Funnel plot and Egg regression will be carried out if >10 qualified studies are included.

### 2.13 Quality of evidence

Grading of Recommendations Assessment, Development and Evaluation approach will be utilized to evaluate the evidence quality for the main outcomes. We will assess 5 items in limitations of study design, inconsistency, inaccuracies, indirectness, and publication bias.

## 3 Discussion

The protocol of this systematic review will assess the efficacy and safety of LMWH monotherpay for the treatment of patients with RCA and APS. To our best knowledge, no systematic review has specifically addressed this issue although several high quality trials have been published.^[13,16–22]^ Thus, it is very important and very necessary to conduct this systematic review to investigate the efficacy and safety of LMWH for RCA with APS.

In this systematic review, we will try our best to search comprehensive literatures without language restrictions. All potential studies regarding LMWH for RCA with APS will be fully considered to avoid missing any potential trials. The results of this systematic review will provide a summary of latest evidence on the efficacy and safety of LMWH for RCA with APS.

## Author contributions

**Conceptualization:** Lei Ni, Dong-xu Zhao, Zi-wei Zhu.

**Data curation:** Lei Ni, Xue-qian Sun, Zi-wei Zhu.

**Formal analysis:** Xue-qian Sun, Dong-xu Zhao, Zi-wei Zhu.

**Funding acquisition:** Lei Ni.

**Investigation:** Lei Ni, Zi-wei Zhu.

**Methodology:** Lei Ni, Xue-qian Sun, Dong-xu Zhao, Zi-wei Zhu.

**Project administration:** Lei Ni.

**Resources:** Xue-qian Sun, Dong-xu Zhao, Zi-wei Zhu.

**Software:** Xue-qian Sun, Dong-xu Zhao, Zi-wei Zhu.

**Supervision:** Lei Ni.

**Validation:** Lei Ni, Xue-qian Sun, Dong-xu Zhao, Zi-wei Zhu.

**Visualization:** Lei Ni, Xue-qian Sun, Dong-xu Zhao.

**Writing – original draft:** Lei Ni.

**Writing – review & editing:** Lei Ni, Xue-qian Sun, Dong-xu Zhao, Zi-wei Zhu.
